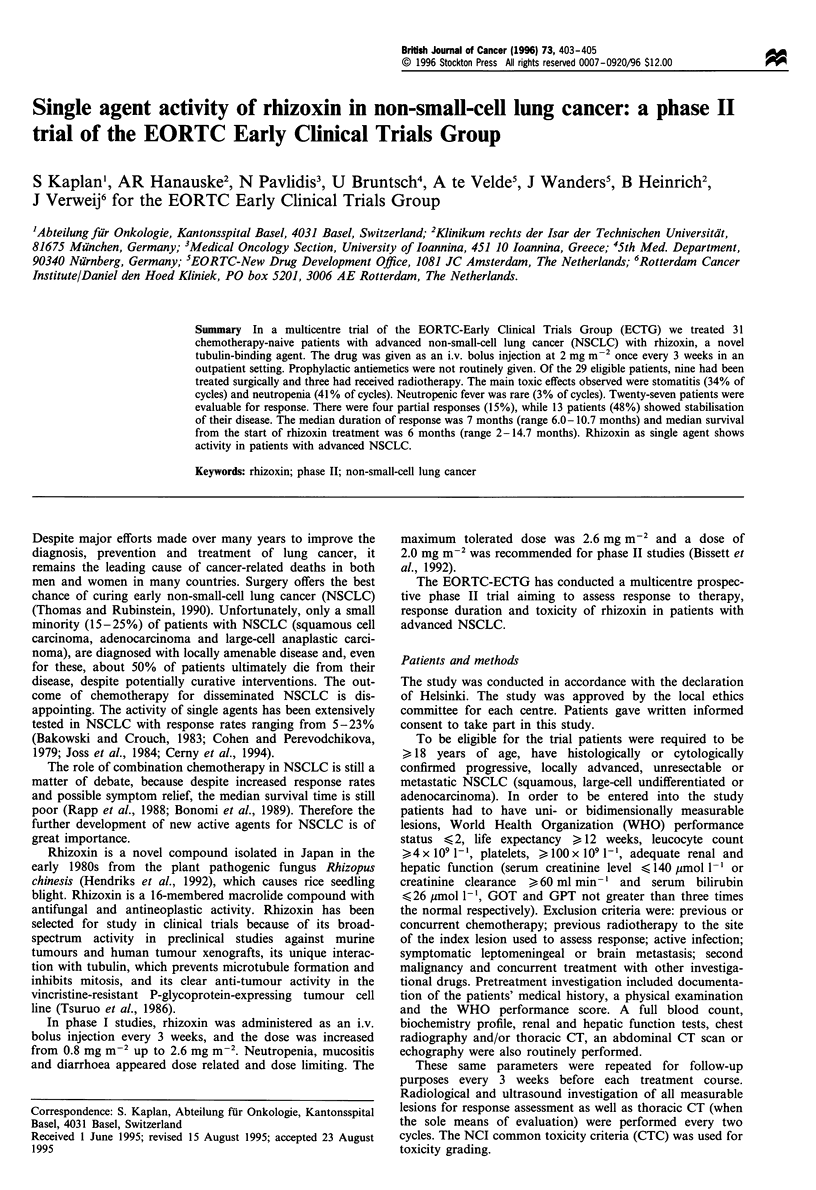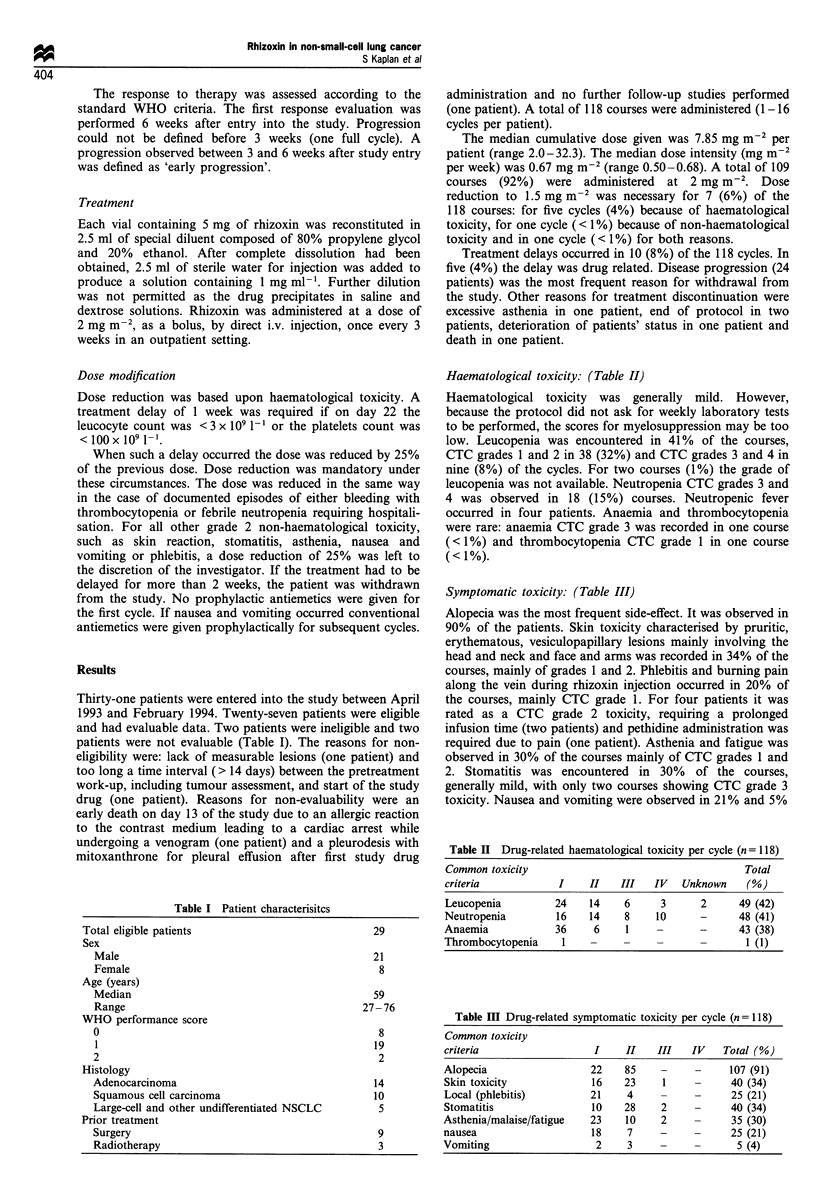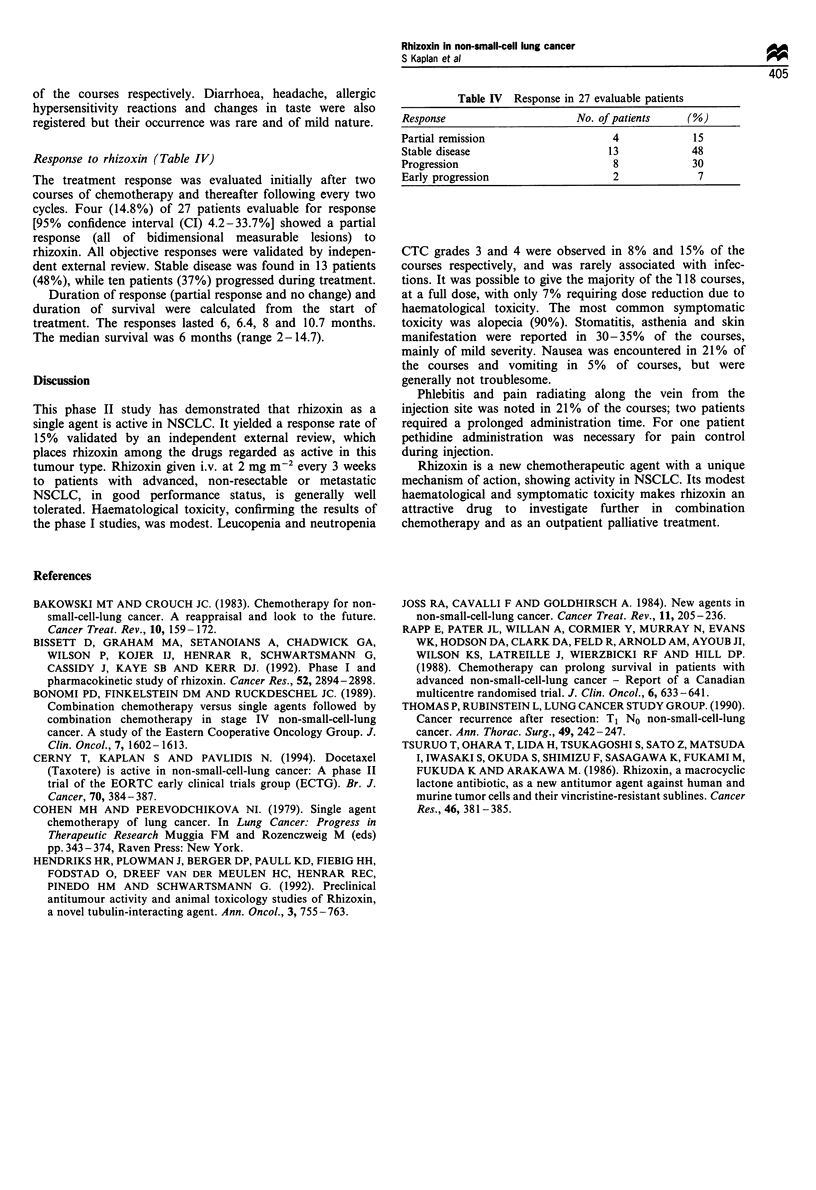# Single agent activity of rhizoxin in non-small-cell lung cancer: a phase II trial of the EORTC Early Clinical Trials Group.

**DOI:** 10.1038/bjc.1996.70

**Published:** 1996-02

**Authors:** S. Kaplan, A. R. Hanauske, N. Pavlidis, U. Bruntsch, A. te Velde, J. Wanders, B. Heinrich, J. Verweij

**Affiliations:** Abteilung für Onkologie, Kantonsspital Basel, Switzerland.

## Abstract

In a multicentre trial of the EORTC-Early Clinical Trials Group (ECTG) we treated 31 chemotherapy-naive patients with advanced non-small-cell lung cancer (NSCLC) with rhizoxin, a novel tubulin-binding agent. The drug was given as an i.v. bolus injection at 2 mg m-2 once every 3 weeks in an outpatient setting. Prophylactic antiemetics were not routinely given. Of the 29 eligible patients, nine had been treated surgically and three had received radiotherapy. The main toxic effects observed were stomatitis (34% of cycles) and neutropenia (41% of cycles). Neutropenic fever was rare (3% of cycles). Twenty-seven patients were evaluable for response. There were four partial responses (15%), while 13 patients (48%) showed stabilisation of their disease. The median duration of response was 7 months (range 6.0-10.7 months) and median survival from the start of rhizoxin treatment was 6 months (range 2-14.7 months). Rhizoxin as single agent shows activity in patients with advanced NSCLC.


British Journal of Cancer (1996) 73, 403-405
© 1996 Stockton Press All rights reserved 0007-0920/96 $12.00

### Single agent activity of rhizoxin in non-small-cell lung cancer: a phase II trial of the EORTC Early Clinical Trials Group

S Kaplan[1], AR Hanauske[2], N Pavlidis[3], U Bruntsch[4], A te Velde[5], J Wanders[5], B Heinrich[2], J Verweij[6] for the EORTC Early Clinical Trials Group

[1]*Abteilung für Onkologie, Kantonsspital Basel, 4031 Basel, Switzerland;* [2]*Klinikum rechts der Isar der Technischen Universität, 81675 München, Germany;* [3]*Medical Oncology Section, University of Ioannina, 451 10 Ioannina, Greece;* [4]*5th Med. Department, 90340 Nürnberg, Germany;* [5]*EORTC-New Drug Development Office, 1081 JC Amsterdam, The Netherlands;* [6]*Rotterdam Cancer Institute/Daniel den Hoed Kliniek, PO box 5201, 3006 AE Rotterdam, The Netherlands.*

**Summary** In a multicentre trial of the EORTC-Early Clinical Trials Group (ECTG) we treated 31 chemotherapy-naive patients with advanced non-small-cell lung cancer (NSCLC) with rhizoxin, a novel tubulin-binding agent. The drug was given as an i.v. bolus injection at 2 mg $m^{-2}$ once every 3 weeks in an outpatient setting. Prophylactic antiemetics were not routinely given. Of the 29 eligible patients, nine had been treated surgically and three had received radiotherapy. The main toxic effects observed were stomatitis (34% of cycles) and neutropenia (41% of cycles). Neutropenic fever was rare (3% of cycles). Twenty-seven patients were evaluable for response. There were four partial responses (15%), while 13 patients (48%) showed stabilisation of their disease. The median duration of response was 7 months (range 6.0-10.7 months) and median survival from the start of rhizoxin treatment was 6 months (range 2-14.7 months). Rhizoxin as single agent shows activity in patients with advanced NSCLC.

**Keywords:** rhizoxin; phase II; non-small-cell lung cancer

Despite major efforts made over many years to improve the diagnosis, prevention and treatment of lung cancer, it remains the leading cause of cancer-related deaths in both men and women in many countries. Surgery offers the best chance of curing early non-small-cell lung cancer (NSCLC) (Thomas and Rubinstein, 1990). Unfortunately, only a small minority (15-25%) of patients with NSCLC (squamous cell carcinoma, adenocarcinoma and large-cell anaplastic carcinoma), are diagnosed with locally amenable disease and, even for these, about 50% of patients ultimately die from their disease, despite potentially curative interventions. The outcome of chemotherapy for disseminated NSCLC is disappointing. The activity of single agents has been extensively tested in NSCLC with response rates ranging from 5-23% (Bakowski and Crouch, 1983; Cohen and Perevodchikova, 1979; Joss *et al.*, 1984; Cerny *et al.*, 1994).

The role of combination chemotherapy in NSCLC is still a matter of debate, because despite increased response rates and possible symptom relief, the median survival time is still poor (Rapp *et al.*, 1988; Bonomi *et al.*, 1989). Therefore the further development of new active agents for NSCLC is of great importance.

Rhizoxin is a novel compound isolated in Japan in the early 1980s from the plant pathogenic fungus *Rhizopus chinesis* (Hendriks *et al.*, 1992), which causes rice seedling blight. Rhizoxin is a 16-membered macrolide compound with antifungal and antineoplastic activity. Rhizoxin has been selected for study in clinical trials because of its broad-spectrum activity in preclinical studies against murine tumours and human tumour xenografts, its unique interaction with tubulin, which prevents microtubule formation and inhibits mitosis, and its clear anti-tumour activity in the vincristine-resistant P-glycoprotein-expressing tumour cell line (Tsuruo *et al.*, 1986).

In phase I studies, rhizoxin was administered as an i.v. bolus injection every 3 weeks, and the dose was increased from 0.8 mg $m^{-2}$ up to 2.6 mg $m^{-2}$. Neutropenia, mucositis and diarrhoea appeared dose related and dose limiting. The maximum tolerated dose was 2.6 mg $m^{-2}$ and a dose of 2.0 mg $m^{-2}$ was recommended for phase II studies (Bissett *et al.*, 1992).

The EORTC-ECTG has conducted a multicentre prospective phase II trial aiming to assess response to therapy, response duration and toxicity of rhizoxin in patients with advanced NSCLC.

### *Patients and methods*

The study was conducted in accordance with the declaration of Helsinki. The study was approved by the local ethics committee for each centre. Patients gave written informed consent to take part in this study.

To be eligible for the trial patients were required to be $\geqslant 18$ years of age, have histologically or cytologically confirmed progressive, locally advanced, unresectable or metastatic NSCLC (squamous, large-cell undifferentiated or adenocarcinoma). In order to be entered into the study patients had to have uni- or bidimensionally measurable lesions, World Health Organization (WHO) performance status $\leqslant 2$, life expectancy $\geqslant 12$ weeks, leucocyte count $\geqslant 4 \times 10^9$ $l^{-1}$, platelets, $\geqslant 100 \times 10^9$ $l^{-1}$, adequate renal and hepatic function (serum creatinine level $\leqslant 140$ $\mu$mol $l^{-1}$ or creatinine clearance $\geqslant 60$ ml $min^{-1}$ and serum bilirubin $\leqslant 26$ $\mu$mol $l^{-1}$, GOT and GPT not greater than three times the normal respectively). Exclusion criteria were: previous or concurrent chemotherapy; previous radiotherapy to the site of the index lesion used to assess response; active infection; symptomatic leptomeningeal or brain metastasis; second malignancy and concurrent treatment with other investigational drugs. Pretreatment investigation included documentation of the patients' medical history, a physical examination and the WHO performance score. A full blood count, biochemistry profile, renal and hepatic function tests, chest radiography and/or thoracic CT, an abdominal CT scan or echography were also routinely performed.

These same parameters were repeated for follow-up purposes every 3 weeks before each treatment course. Radiological and ultrasound investigation of all measurable lesions for response assessment as well as thoracic CT (when the sole means of evaluation) were performed every two cycles. The NCI common toxicity criteria (CTC) was used for toxicity grading.

Correspondence: S. Kaplan, Abteilung für Onkologie, Kantonsspital Basel, 4031 Basel, Switzerland
Received 1 June 1995; revised 15 August 1995; accepted 23 August 1995

The response to therapy was assessed according to the standard WHO criteria. The first response evaluation was performed 6 weeks after entry into the study. Progression could not be defined before 3 weeks (one full cycle). A progression observed between 3 and 6 weeks after study entry was defined as 'early progression'.

*Treatment*

Each vial containing 5 mg of rhizoxin was reconstituted in 2.5 ml of special diluent composed of 80% propylene glycol and 20% ethanol. After complete dissolution had been obtained, 2.5 ml of sterile water for injection was added to produce a solution containing 1 mg $ml^{-1}$. Further dilution was not permitted as the drug precipitates in saline and dextrose solutions. Rhizoxin was administered at a dose of 2 mg $m^{-2}$, as a bolus, by direct i.v. injection, once every 3 weeks in an outpatient setting.

*Dose modification*

Dose reduction was based upon haematological toxicity. A treatment delay of 1 week was required if on day 22 the leucocyte count was $<3\times10^9\ l^{-1}$ or the platelets count was $<100\times10^9\ l^{-1}$.

When such a delay occurred the dose was reduced by 25% of the previous dose. Dose reduction was mandatory under these circumstances. The dose was reduced in the same way in the case of documented episodes of either bleeding with thrombocytopenia or febrile neutropenia requiring hospitalisation. For all other grade 2 non-haematological toxicity, such as skin reaction, stomatitis, asthenia, nausea and vomiting or phlebitis, a dose reduction of 25% was left to the discretion of the investigator. If the treatment had to be delayed for more than 2 weeks, the patient was withdrawn from the study. No prophylactic antiemetics were given for the first cycle. If nausea and vomiting occurred conventional antiemetics were given prophylactically for subsequent cycles.

### Results

Thirty-one patients were entered into the study between April 1993 and February 1994. Twenty-seven patients were eligible and had evaluable data. Two patients were ineligible and two patients were not evaluable (Table I). The reasons for non-eligibility were: lack of measurable lesions (one patient) and too long a time interval (>14 days) between the pretreatment work-up, including tumour assessment, and start of the study drug (one patient). Reasons for non-evaluability were an early death on day 13 of the study due to an allergic reaction to the contrast medium leading to a cardiac arrest while undergoing a venogram (one patient) and a pleurodesis with mitoxanthrone for pleural effusion after first study drug administration and no further follow-up studies performed (one patient). A total of 118 courses were administered (1–16 cycles per patient).

The median cumulative dose given was 7.85 mg $m^{-2}$ per patient (range 2.0–32.3). The median dose intensity (mg $m^{-2}$ per week) was 0.67 mg $m^{-2}$ (range 0.50–0.68). A total of 109 courses (92%) were administered at 2 mg $m^{-2}$. Dose reduction to 1.5 mg $m^{-2}$ was necessary for 7 (6%) of the 118 courses: for five cycles (4%) because of haematological toxicity, for one cycle (<1%) because of non-haematological toxicity and in one cycle (<1%) for both reasons.

Treatment delays occurred in 10 (8%) of the 118 cycles. In five (4%) the delay was drug related. Disease progression (24 patients) was the most frequent reason for withdrawal from the study. Other reasons for treatment discontinuation were excessive asthenia in one patient, end of protocol in two patients, deterioration of patients' status in one patient and death in one patient.

*Haematological toxicity: (Table II)*

Haematological toxicity was generally mild. However, because the protocol did not ask for weekly laboratory tests to be performed, the scores for myelosuppression may be too low. Leucopenia was encountered in 41% of the courses, CTC grades 1 and 2 in 38 (32%) and CTC grades 3 and 4 in nine (8%) of the cycles. For two courses (1%) the grade of leucopenia was not available. Neutropenia CTC grades 3 and 4 was observed in 18 (15%) courses. Neutropenic fever occurred in four patients. Anaemia and thrombocytopenia were rare: anaemia CTC grade 3 was recorded in one course (<1%) and thrombocytopenia CTC grade 1 in one course (<1%).

*Symptomatic toxicity: (Table III)*

Alopecia was the most frequent side-effect. It was observed in 90% of the patients. Skin toxicity characterised by pruritic, erythematous, vesiculopapillary lesions mainly involving the head and neck and face and arms was recorded in 34% of the courses, mainly of grades 1 and 2. Phlebitis and burning pain along the vein during rhizoxin injection occurred in 20% of the courses, mainly CTC grade 1. For four patients it was rated as a CTC grade 2 toxicity, requiring a prolonged infusion time (two patients) and pethidine administration was required due to pain (one patient). Asthenia and fatigue was observed in 30% of the courses mainly of CTC grades 1 and 2. Stomatitis was encountered in 30% of the courses, generally mild, with only two courses showing CTC grade 3 toxicity. Nausea and vomiting were observed in 21% and 5%

**Table I** Patient characterisitcs

| | |
|---|---|
| Total eligible patients | 29 |
| Sex | |
| Male | 21 |
| Female | 8 |
| Age (years) | |
| Median | 59 |
| Range | 27–76 |
| WHO performance score | |
| 0 | 8 |
| 1 | 19 |
| 2 | 2 |
| Histology | |
| Adenocarcinoma | 14 |
| Squamous cell carcinoma | 10 |
| Large-cell and other undifferentiated NSCLC | 5 |
| Prior treatment | |
| Surgery | 9 |
| Radiotherapy | 3 |

**Table II** Drug-related haematological toxicity per cycle ($n=118$)

| *Common toxicity criteria* | *I* | *II* | *III* | *IV* | *Unknown* | *Total (%)* |
|---|---|---|---|---|---|---|
| Leucopenia | 24 | 14 | 6 | 3 | 2 | 49 (42) |
| Neutropenia | 16 | 14 | 8 | 10 | – | 48 (41) |
| Anaemia | 36 | 6 | 1 | – | – | 43 (38) |
| Thrombocytopenia | 1 | – | – | – | – | 1 (1) |

**Table III** Drug-related symptomatic toxicity per cycle ($n=118$)

| *Common toxicity criteria* | *I* | *II* | *III* | *IV* | *Total (%)* |
|---|---|---|---|---|---|
| Alopecia | 22 | 85 | – | – | 107 (91) |
| Skin toxicity | 16 | 23 | 1 | – | 40 (34) |
| Local (phlebitis) | 21 | 4 | – | – | 25 (21) |
| Stomatitis | 10 | 28 | 2 | – | 40 (34) |
| Asthenia/malaise/fatigue | 23 | 10 | 2 | – | 35 (30) |
| nausea | 18 | 7 | – | – | 25 (21) |
| Vomiting | 2 | 3 | – | – | 5 (4) |

of the courses respectively. Diarrhoea, headache, allergic hypersensitivity reactions and changes in taste were also registered but their occurrence was rare and of mild nature.

*Response to rhizoxin (Table IV)*

The treatment response was evaluated initially after two courses of chemotherapy and thereafter following every two cycles. Four (14.8%) of 27 patients evaluable for response [95% confidence interval (CI) 4.2–33.7%] showed a partial response (all of bidimensional measurable lesions) to rhizoxin. All objective responses were validated by independent external review. Stable disease was found in 13 patients (48%), while ten patients (37%) progressed during treatment.

Duration of response (partial response and no change) and duration of survival were calculated from the start of treatment. The responses lasted 6, 6.4, 8 and 10.7 months. The median survival was 6 months (range 2–14.7).

**Table IV** Response in 27 evaluable patients

| *Response* | *No. of patients* | *(%)* |
|---|---|---|
| Partial remission | 4 | 15 |
| Stable disease | 13 | 48 |
| Progression | 8 | 30 |
| Early progression | 2 | 7 |

### Discussion

This phase II study has demonstrated that rhizoxin as a single agent is active in NSCLC. It yielded a response rate of 15% validated by an independent external review, which places rhizoxin among the drugs regarded as active in this tumour type. Rhizoxin given i.v. at 2 mg $m^{-2}$ every 3 weeks to patients with advanced, non-resectable or metastatic NSCLC, in good performance status, is generally well tolerated. Haematological toxicity, confirming the results of the phase I studies, was modest. Leucopenia and neutropenia CTC grades 3 and 4 were observed in 8% and 15% of the courses respectively, and was rarely associated with infections. It was possible to give the majority of the 118 courses, at a full dose, with only 7% requiring dose reduction due to haematological toxicity. The most common symptomatic toxicity was alopecia (90%). Stomatitis, asthenia and skin manifestation were reported in 30–35% of the courses, mainly of mild severity. Nausea was encountered in 21% of the courses and vomiting in 5% of courses, but were generally not troublesome.

Phlebitis and pain radiating along the vein from the injection site was noted in 21% of the courses; two patients required a prolonged administration time. For one patient pethidine administration was necessary for pain control during injection.

Rhizoxin is a new chemotherapeutic agent with a unique mechanism of action, showing activity in NSCLC. Its modest haematological and symptomatic toxicity makes rhizoxin an attractive drug to investigate further in combination chemotherapy and as an outpatient palliative treatment.

### References

BAKOWSKI MT AND CROUCH JC. (1983). Chemotherapy for non-small-cell-lung cancer. A reappraisal and look to the future. *Cancer Treat. Rev.*, **10,** 159–172.

BISSETT D, GRAHAM MA, SETANOIANS A, CHADWICK GA, WILSON P, KOJER IJ, HENRAR R, SCHWARTSMANN G, CASSIDY J, KAYE SB AND KERR DJ. (1992). Phase I and pharmacokinetic study of rhizoxin. *Cancer Res.*, **52,** 2894–2898.

BONOMI PD, FINKELSTEIN DM AND RUCKDESCHEL JC. (1989). Combination chemotherapy versus single agents followed by combination chemotherapy in stage IV non-small-cell-lung cancer. A study of the Eastern Cooperative Oncology Group. *J. Clin. Oncol.*, **7,** 1602–1613.

CERNY T, KAPLAN S AND PAVLIDIS N. (1994). Docetaxel (Taxotere) is active in non-small-cell-lung cancer: A phase II trial of the EORTC early clinical trials group (ECTG). *Br. J. Cancer*, **70,** 384–387.

COHEN MH AND PEREVODCHIKOVA NI. (1979). Single agent chemotherapy of lung cancer. In *Lung Cancer: Progress in Therapeutic Research* Muggia FM and Rozenczweig M (eds) pp. 343–374, Raven Press: New York.

HENDRIKS HR, PLOWMAN J, BERGER DP, PAULL KD, FIEBIG HH, FODSTAD O, DREEF VAN DER MEULEN HC, HENRAR REC, PINEDO HM AND SCHWARTSMANN G. (1992). Preclinical antitumour activity and animal toxicology studies of Rhizoxin, a novel tubulin-interacting agent. *Ann. Oncol.*, **3,** 755–763.

JOSS RA, CAVALLI F AND GOLDHIRSCH A. 1984). New agents in non-small-cell-lung cancer. *Cancer Treat. Rev.*, **11,** 205–236.

RAPP E, PATER JL, WILLAN A, CORMIER Y, MURRAY N, EVANS WK, HODSON DA, CLARK DA, FELD R, ARNOLD AM, AYOUB JI, WILSON KS, LATREILLE J, WIERZBICKI RF AND HILL DP. (1988). Chemotherapy can prolong survival in patients with advanced non-small-cell-lung cancer – Report of a Canadian multicentre randomised trial. *J. Clin. Oncol.*, **6,** 633–641.

THOMAS P, RUBINSTEIN L, LUNG CANCER STUDY GROUP. (1990). Cancer recurrence after resection: $T_1$ $N_0$ non-small-cell-lung cancer. *Ann. Thorac. Surg.*, **49,** 242–247.

TSURUO T, OHARA T, LIDA H, TSUKAGOSHI S, SATO Z, MATSUDA I, IWASAKI S, OKUDA S, SHIMIZU F, SASAGAWA K, FUKAMI M, FUKUDA K AND ARAKAWA M. (1986). Rhizoxin, a macrocyclic lactone antibiotic, as a new antitumor agent against human and murine tumor cells and their vincristine-resistant sublines. *Cancer Res.*, **46,** 381–385.